# Gut microbiome and metabolic responses to cricket powder supplementation in Thai subjects with high or borderline-high LDL cholesterol: an exploratory, randomized, crossover controlled trial

**DOI:** 10.1016/j.crfs.2026.101536

**Published:** 2026-08-21

**Authors:** Aree Prachansuwan, Pitchayaporn Sukkha, Parunya Thiyajai, Pitakthai Chamtim, Sarunya Kitdumrongthum, Pimnapanut Sridonpai, Angkansiri Dee-uam, Karaked Tongdonpo, Dunyaporn Trachootham, Warangkana Srichamnong, Iyarit Thaipisuttikul, Nachon Raethong

**Affiliations:** a Institute of Nutrition, Mahidol University, Nakhon Pathom, 73170, Thailand; b Master of Science Program in Toxicology and Nutrition for Food Safety, Institute of Nutrition, Mahidol University, Nakhon Pathom, 73170, Thailand; c National Bureau of Agricultural Commodity and Food Standards, Chatuchak, Bangkok, 10900, Thailand; d Academic Service Division, National Laboratory Animal Center, Mahidol University, Nakhon Pathom, 73170, Thailand; e Department of Microbiology, Faculty of Medicine Siriraj Hospital, Mahidol University, Bangkok, 10700, Thailand

**Keywords:** Gut microbiome, Edible insect, Cricket, Prebiotic, Metagenomics

## Abstract

Edible insects are emerging as sustainable functional foods, yet human evidence for microbiome-mediated effects remains limited, particularly in Asian populations. Therefore, this study investigated whether cricket powder supplementation modulates gut microbiome composition and metabolic outputs in Thai subjects with high or borderline-high LDL cholesterol. In a randomized, crossover controlled trial, 17 subjects received cricket powder or control products for 21 days, separated by a 4-week washout. Gut microbiome composition was profiled using full-length 16S rRNA gene sequencing, and fecal short-chain fatty acids (SCFAs) were quantified by gas chromatography-mass spectrometry. Blood lipids and gastrointestinal tolerance were also assessed. As a result, cricket powder did not alter overall microbial diversity or community structure but induced targeted species-level shifts, including enrichment of *Blautia faecis* and *Mediterraneibacter glycyrrhizinilyticus*. Despite these compositional changes, fecal SCFAs remained unchanged. Notably, branched-chain SCFAs were not increased, indicating no shift toward proteolytic fermentation and preservation of microbial metabolic balance. Gastrointestinal tolerance was maintained without adverse effects. Blood lipid parameters were unchanged, with a modest trend toward increased high-density lipoprotein cholesterol (HDL-C). Collectively, cricket powder induces selective microbiome remodeling without disrupting metabolic homeostasis, supporting its potential as a sustainable, microbiome-targeted functional food.

## Highlights

• Cricket powder drives targeted species-level microbiome remodeling.
• SCFA balance is preserved without proteolytic fermentation.
• Cricket powder is well tolerated in adults with high or borderline-high LDL cholesterol.
• HDL-C trends upward after cricket powder supplementation.

## Graphical abstract

**Figure:**
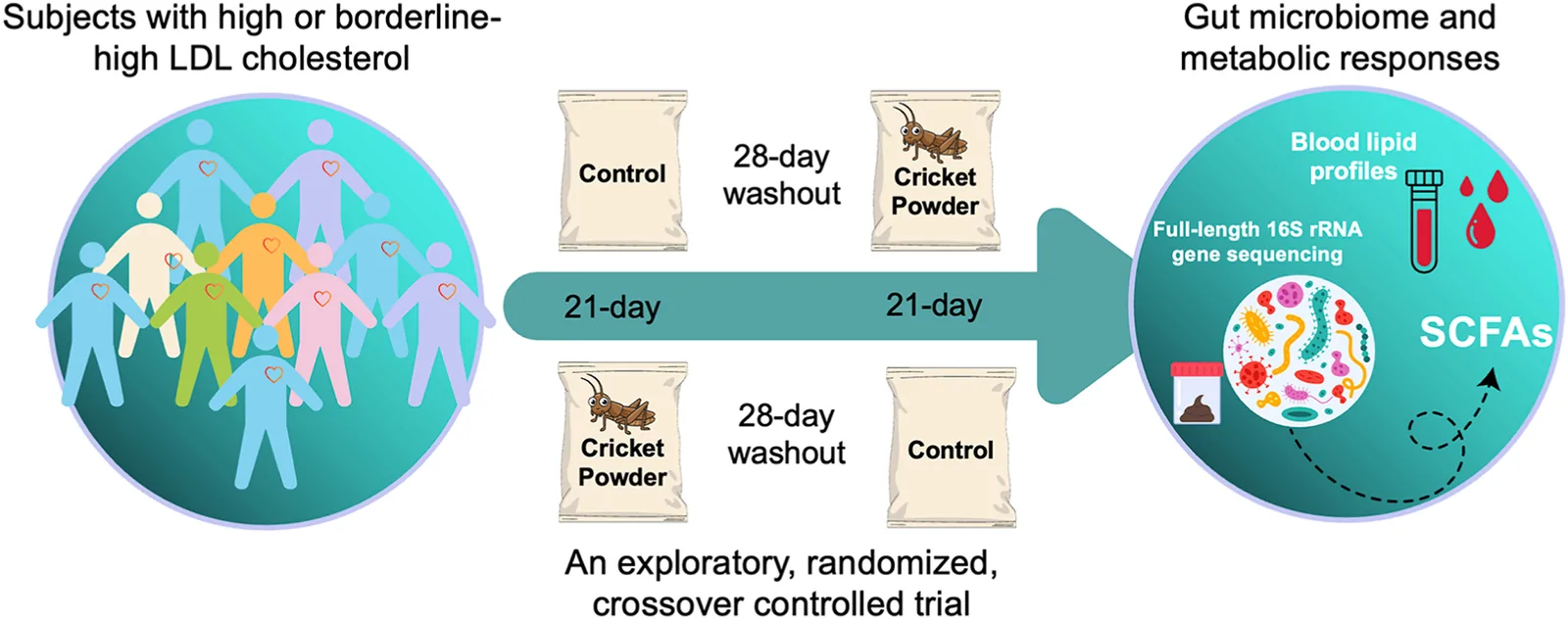

## 1 Introduction

Cardiovascular disease (CVD) is responsible for more than 17 million deaths worldwide (World Health Organization (WHO), 2021). CVD is associated with hypercholesterolemia by mediation of atherosclerosis, which results from lipid accumulation in arterial walls and leads to myocardial infarction and stroke (Bays et al., 2022). Diet is crucial, and gut dysbiosis is linked to hypercholesterolemia (Lei et al., 2022).

Gut microbiome describes the group of microorganisms and their functions that are created the interactions between the host, gut bacteria, and food ingredients (Raethong et al., 2026). Comprising trillions of microorganisms, the gut microbiome is essential for the conversion of exogenous drugs, host-secreted chemicals, and dietary substrates into bioactive metabolites, essential for controlling host metabolism, immunological response, and general health (Nicholson et al., 2012; Koh et al., 2016). Dietary interventions promoting beneficial gut bacteria and supporting gastrointestinal and metabolic functions represent a rational strategy for targeting hypercholesterolemia (O'Riordan et al., 2022). Responses of the gut microbiome to dietary components, including probiotics, prebiotics, and fiber-rich foods, have been shown to influence human physiology, through the production and exchange of metabolites (Hindle et al., 2025). Butyrate, acetate, and propionate, are specifically gut microbial short-chain fatty acids (SCFAs) that have been found to exhibit various positive effects, including ameliorating the development of hypercholesterolemia (Arboleya et al., 2016). A previous pre-clinical investigations showed that low-density lipoprotein cholesterol (LDL-C) and total triglyceride levels dropped by alteration of gut microbiome (Balaguer et al., 2022; Velagapudi et al., 2010).

In addition to indicating potential metabolic benefits, changes in gut microbiome composition and microbial metabolites may provide complementary information on gastrointestinal safety and intestinal homeostasis. For example, subacute oral toxicity evaluation of copra meal hydrolysate, a novel prebiotic rich in mannooligosaccharides, demonstrated that gut microbiome composition and intestinal parameter analysis confirmed its high safety profile without inducing adverse tissue or histological changes in Sprague Dawley rats (Tangthong et al., 2025). Similarly, a randomized, double-blind trial showed that high-protein diets shifted bacterial metabolism toward amino acid degradation and altered rectal mucosal homeostasis, despite not inducing overt intestinal inflammation or fecal water cytotoxicity (Beaumont et al., 2017). Collectively, evaluating gut microbiome composition and SCFA production following the consumption of a protein-rich food may help identify beneficial, neutral, or potentially unfavorable intestinal responses, thereby complementing conventional clinical safety assessments.

Global food insecurity and the increasing demand for sustainable, nutrient-dense food sources have driven interest in alternative proteins such as edible insects. Among these, crickets are attractive because they provide protein, fatty acids, and micronutrients (Kemsawasd et al., 2022). Crickets are also rich in chitin and chitin derivatives, which can inhibit the growth of pathogenic bacteria, without affecting health-promoting bacteria (Imathiu, 2020). With historical practice of consuming crickets in Asia, cricket is safe with respect to nutritional composition, pesticide levels, heavy metals, and microorganisms (EFSA Panel on Nutrition et al., 2022). Beyond their nutritional benefits and safety profiles, recent human study has investigated the effects of whole cricket powder on gut microbiome. A previous study by Stull et al. (2018) showed that whole cricket powder increased the abundance of SCFA-producing bacteria by up to 5.7-fold (Stull et al., 2018). While cricket has not raised major safety concerns, cricket may exert prebiotic functions by modulating the growth of SCFA-producing bacteria, which may contribute to the prevention of hypercholesterolemia (Arboleya et al., 2016; Stull et al., 2018).

However, one of the challenges in establishing the prebiotic function of cricket is that gut microbiome responses are not consistent across global populations. Dietary background strongly influences gut microbiome structure, particularly between Asian and Western populations (Vangay et al., 2018). Moreover, habitual diets differ across populations, for example, Thai adults have an average daily intake of cricket of 22.26 ± 4.45 g/day (Raethong et al., 2026). In addition, the functional benefits of cricket consumption and the metabolic implications of cricket-induced microbial shifts, particularly in relation to lipid metabolism, remain limited. Currently, most available evidence has been generated in non-Asian and healthy populations. Additionally, gastrointestinal safety and intestinal homeostasis, as reflected by gut microbiome responses following cricket consumption, have not been adequately investigated.

In the present study, we assessed gut microbiome responses to cricket powder supplementation through a randomized, crossover controlled trial in Thai subjects with high or borderline-high LDL-C. The primary endpoints were changes in gut microbiome composition, fecal SCFA concentrations, gastrointestinal symptoms, and tolerance after 21 consecutive days of intervention. Dietary intake and compliance were also evaluated. To assess the potential contribution of the microbiome to metabolic outcomes, blood lipid profiles were analyzed. These findings may contribute to the development of sustainable, functional, and health-promoting dietary strategies, while supporting the broader integration of cricket-based products as prebiotics in future food systems.

## 2 Methods

This study was reviewed and approved by the Mahidol University Central Institutional Review Board (MU-CIRB; COA No. MU-MOU 2023/334.2710) and registered at ClinicalTrials.gov (identifier: NCT07009756). All subjects provided written informed consent before beginning the study. The study was conducted in accordance with the principles of the Declaration of Helsinki.

### 2.1 Subjects and study design

This study was a randomized, crossover controlled trial. Partially detailed information was given in our previous publication (Sukkha et al., 2025). The enrollment of subjects was between March and June 2024. The eligibility criteria were as follows: men and women aged 20 - 60 years with a body mass index (BMI) of 18.5 - 29.9 kg/m^2^ and high or borderline-high LDL-C, defined as fasting total cholesterol >200 mg/dL and LDL-C of 130 - 189 mg/dL according to the NCEP-ATP III guidelines (Expert Panel on Detection and Evaluation, 2001). Additional inclusion criteria included fasting triglycerides within the normal to high range (<500 mg/dL), non-diabetic status (fasting plasma glucose <110 mg/dL), normal liver and kidney function (ALT and AST <60 U/L and estimated glomerular filtration rate (eGFR) ≥90 mL/min/1.73 m^2^). Subjects were also required to have controlled blood pressure (systolic blood pressure <160 mmHg, diastolic blood pressure <90 mmHg, and heart rate <100 bpm), and a self-reported non-smoking status. The exclusion criteria included allergy to insects, chitin, chitosan, shrimp, dust mites, or other crustacean products; pregnancy or breastfeeding; participation in other research projects; use of drugs, herbs, supplements, steroids, antibiotics, or medications that may affect lipid metabolism; regular consumption of probiotic products that could not be discontinued; and a history of diabetes, chronic liver disease, chronic kidney disease, cardiovascular disease, cancer, anemia, irritable bowel syndrome, ulcerative colitis, or anaphylaxis.

Sample size was calculated with 80% power, a two-sided alpha of 0.05, and an effect size of 0.827 based on *Ruminococcaceae*_UCG-005 data from previous study (Ranaivo et al., 2022). The required sample size was 15 subjects per group, which was increased to 20 per group to account for a 25% dropout rate.

### 2.2 Intervention and assessment

Initially, for the first intervention phase, subjects were randomized to intervention group or control group. Randomization was stratified by sex, and a minimization procedure was used to allocate subjects. After the first intervention phase (3 weeks), a washout period (4 weeks) and the second crossover intervention phase (3 weeks) followed. For intervention group, subjects were received one daily serving of cricket powder. Each serving contained 21.5 g of house cricket (*Acheta domesticus*) powder, purchased from Protanica Co., LTD., Thailand. The estimated energy and nutrient composition of cricket powder, calculated per 100 g, is presented in Table 1. The cricket powder was prepared under sterile conditions and heat-treated to ensure microbiological safety in accordance with Thai public health regulations, as described previously (Sukkha et al., 2025). Each sachet of cricket powder was consumed together with 32 g of instant porridge as the food base. For control group, subjects consumed one daily serving of 32 g of instant porridge without cricket powder. The total energy, and macronutrient contents of the intervention and control products, per serving are shown in Table S1. For supplementation procedures, subjects were instructed to prepare the intervention and control products by adding ∼250 mL of hot water (∼100 °C) to the porridge powder and stirring thoroughly until well mixed. The prepared porridge was left to stand for approximately 2 min before consumption. During each intervention period, subjects were asked to consume one serving of the assigned product daily, preferably at a consistent time of day throughout the study.

**Table 1:** Estimated energy and nutrient composition of cricket powder per 100 g.

Energy and nutrient composition per 100 g	Cricket powder
Energy (kcal)	483.65
Total fat (g)	18.14
Total protein (g)	66.51
Total carbohydrate (g)	15.35

Subjects were instructed to maintain a consistent dietary pattern across all study periods, aiming to consume similar types and amounts of foods in each period. Subjects recorded daily intake of the intervention products, including the amount consumed and any missed doses. Compliance, allergy and adverse effect record were monitored weekly throughout the study. Dietary intake was assessed using 3-day dietary records, including two weekdays and one weekend day, and analyzed using the INMUCAL-Nutrients software version 4.0. Stool characteristics were assessed daily using a 7-day stool record, including defecation frequency, stool amount, color, odor, and Bristol Stool Form Scale classification. Physical activity levels were assessed using the Global Physical Activity Questionnaire and expressed activity intensity in metabolic equivalents of tasks (MET-minutes/week).

### 2.3 Fecal sample collection

A total of four fecal samples for gut microbiome and SCFA analyses were collected per subjects at four scheduled visits. The first sample before first intervention phase was collected within 14 days after enrollment. Subjects were instructed to collect fecal samples at home using sterile containers provided by the study, immediately place them in an insulated bag with ice packs, and deliver them to the Institute of Nutrition, Mahidol University on the scheduled visit day. After the first intervention phase, the second sample was collected on day 21 (±2 days). Following a 28-day washout period, a third sample was collected on day 49 (±2 days), and the fourth sample was collected after the second cross-over intervention on day 70 (±2 days). Samples were aliquoted and stored in −80 °C when received in the laboratory.

### 2.4 DNA isolation and full length 16S rRNA gene sequencing analysis

For gut microbiome analysis, aliquoted fecal samples stored at −80 °C were thawed on ice and transferred into 1.5 mL microcentrifuge tubes for DNA extraction using the QIAamp DNA Stool Mini Kit (Qiagen, Germany) according to the manufacturer's instructions. The extracted DNA was amplified targeting the 16S rRNA gene using PCR with repliQa HiFi ToughMix (Quantabio, USA). Approximately 200 ng of PCR product was used for library preparation with a ligation sequencing amplicon-native barcoding kit (Oxford Nanopore Technologies; ONT, Oxford, UK). Full-length 16S rRNA gene sequencing was performed on a Nanopore platform using default settings.

### 2.5 Fecal SFCA analysis

Fecal SCFAs, including acetic acid, propionic acid, butyric acid, isobutyric acid, and isovaleric acid, were quantified using gas chromatography-mass spectrometry (GC-MS), as described by Thiyajai et al. (2025) (Thiyajai et al., 2025). Briefly, for extraction, approximately 20 ± 1.5 mg of fecal material was weighed into a 1.5 mL microcentrifuge tube. The sample was mixed with sodium hydroxide, sodium acetate-d3 (as an internal standard), and methanol and then homogenized with glass beads by vortexed for 30 s. Subsequently, methanol in water was added, followed by vortexing. The samples were then sonicated in an ice bath for 10 min and stored at −20 °C for 30 min. After incubation, samples were centrifuged at 14,000 rpm for 10 min at 4 °C. Seven-hundred microliter of supernatant was transferred to a new tube and evaporated to dryness at 37 °C using a vacuum concentrator.

The dried extract was derivatized with methoxyamine hydrochloride (MeOX) and N-methyl-N-(tert-butyldimethylsilyl) trifluoroacetamide (MTBSTFA). The samples were vortexed and centrifuged again at 14,000 rpm for 10 min at 4 °C. Prior to GC-MS analysis, the supernatant was diluted 10-fold with pyridine. SCFA standard solutions, including acetic acid, propionic acid, butyric acid, isobutyric acid, and isovaleric acid, were freshly prepared and serially diluted into eight concentration levels to generate calibration curves. The standards were dried and derivatized using the same protocol as the fecal samples. Pooled fecal samples were included as quality controls to ensure analytical consistency and reproducibility.

SCFA measurements were performed using an Agilent GC 8890 system coupled with a 7000D GC/TQ mass spectrometer (Agilent Technologies, Santa Clara, CA, USA) and an HP-5MS UI capillary column (15 m × 250 μm i.d., 0.25 μm film thickness; Agilent, Folsom, CA, USA), with helium as the carrier gas at a flow rate of 1.8 mL/min. Samples were injected at 260 °C with a 10:1 split ratio, and separation was achieved using a 27-min oven temperature program range from 70 to 300 °C. Electron ionization was performed at 70 eV, and quantification was conducted in selected ion monitoring (SIM) mode. Data acquisition and calibration curve analysis were performed using Agilent MassHunter Quantitative Analysis software.

To normalize SCFA concentrations to dry matter, fecal moisture content was measured using a moisture analyzer (Sartorius MA35, Germany) using automatic mode. Final SCFA concentrations were expressed as μmol/g fecal dry matter.

### 2.6 Blood sampling and analyses

Blood samples were collected by a registered nurse at four scheduled visits. At each visit, subjects were drawn blood of 2 teaspoons (8 mL) from the antecubital vein. After blood collection, food and beverages were provided to the subjects. The blood was collected in insulated containers and kept at 4 °C for laboratory work. Blood testing measured total cholesterol, LDL-C, HDL-C, triglyceride, and fasting plasma glucose. Total cholesterol, LDL-C, and HDL-C concentrations in plasma were determined using enzymatic approach. Triglyceride levels were measured using the glycerol phosphate oxidase-4-aminoantipyrin (GPO-PAP) method and fasting plasma glucose was determined via the glucose oxidase-4-aminoantipyrine (GOD-PAP) method. In addition, body weight, height, and resting blood pressure were measured at each visit.

### 2.7 Statistical analysis

Statistical analysis was performed using SPSS software version 19 (SPSS Inc., Chicago, IL, USA). The normality of data distribution was assessed using the Shapiro-Wilk test. Continuous variables were expressed as mean ± standard deviation (SD) for normally distributed data, or as median and interquartile range (IQR) for non-normally distributed data. Categorical variables were reported as frequencies and percentages. Within-group difference between pre- and post-treatment were analyzed using paired *t*-test for parametric data or the Wilcoxon signed-rank test for non-parametric data. Moreover, between-group differences in changes (pre-to-post) were compared using the independent *t*-test for parametric data or the Mann-Whitney *U* test for non-parametric data. Statistical significance was set at *p*-value < 0.05.

For the gut microbiome analysis, raw sequencing data were base-called and demultiplexed using Dorado software (Oxford Nanopore Technologies). Primer and adapter sequences were removed using porechop-abi (Bonenfant et al., 2022). Reads were then filtered using Chopper to retain sequences with lengths between 1200 and 1800 base pairs and an average quality score above 10 (with a minimum Q-score of 9). Taxonomic classification at the species level was performed using the Emu pipeline (Curry et al., 2022), and abundance tables were generated for analysis. Alpha diversity and beta diversity of gut microbiome were analyzed using R version 4.5.2 (2025-10-31).

## 3 Results

### 3.1 Subject characteristics and dietary intake

**Fig. 1:**
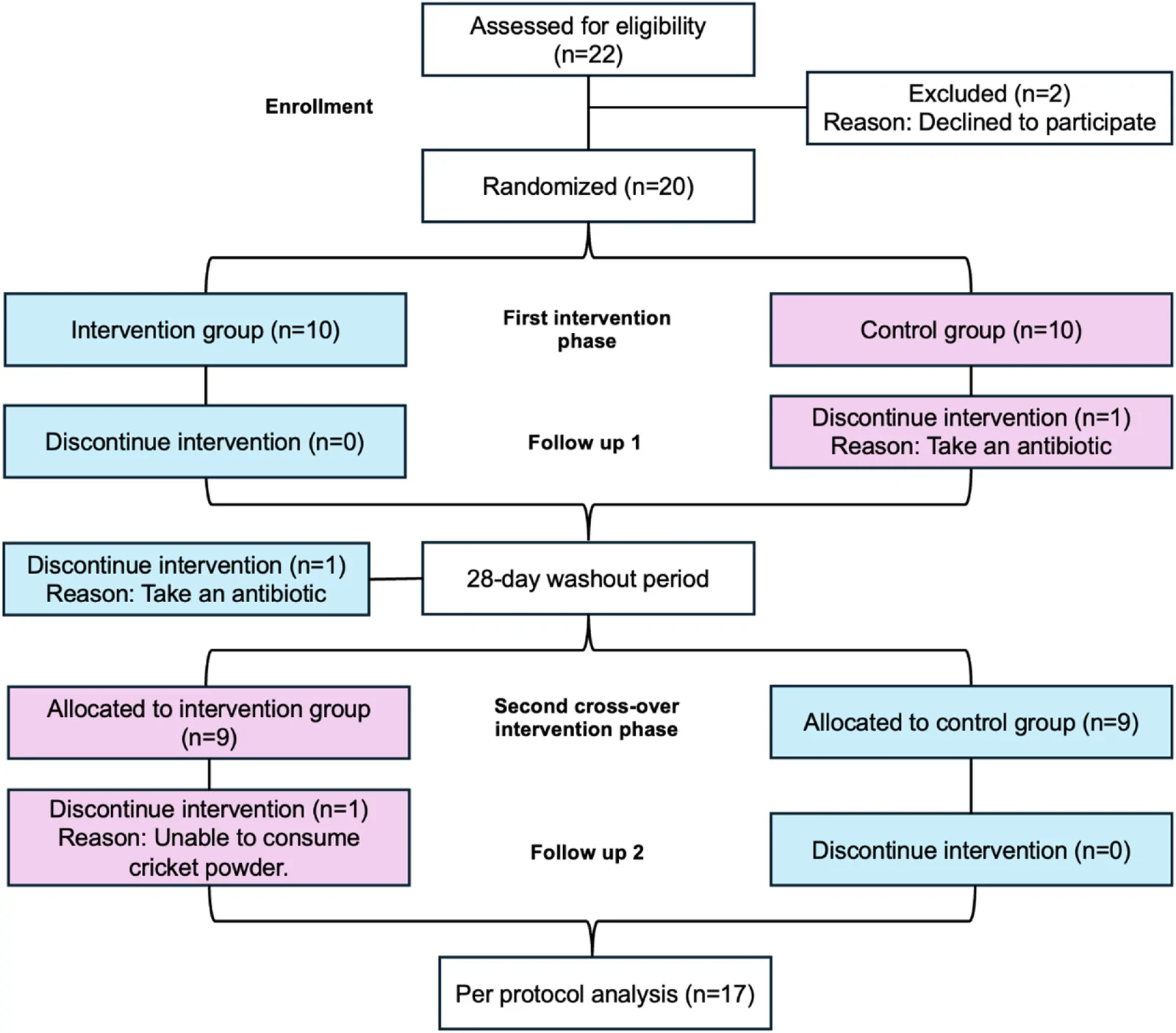
A CONSORT diagram.

A CONSORT diagram detailing the flow of subjects at different stages of the study is shown in Fig. 1. Seventeen completed the crossover study (four men and thirteen women) and were analyzed. Characteristics of study subjects at enrollment are summarized in Table 2. Subjects were adults, with a mean age of 44.65 ± 7.91 years and a mean BMI of 24.12 ± 2.99 kg/m^2^. Fasting plasma glucose levels were within the normal range, with a mean value of 98.18 ± 7.28 mg/dL. All subjects had elevated total cholesterol levels (>200 mg/dL), with a mean total cholesterol of 226.65 ± 26.34 mg/dL, and a mean LDL-C of 155.88 ± 23.41 mg/dL.

**Table 2:** Characteristics of the study subjects at enrollment (n = 17).

Parameters	Value
**Anthropometrics**	
Age (years)	44.65 ± 7.91
Weight (kg)	64.25 ± 8.84
Height (cm)	163.23 ± 7.01
BMI (kg/m^2^)	24.12 ± 2.99
**Blood pressure**	
Systolic blood pressure (mmHg)	123.76 ± 23.41
Diastolic blood pressure (mmHg)	76.59 ± 12.20
**Biochemical parameters**	
Fasting plasma glucose (mg/dL)	98.18 ± 7.28
Total cholesterol (mg/dL)	226.65 ± 26.34
Triglyceride (mg/dL)	94.00 (81.50, 140.50)
HDL-C (mg/dL)	56.24 ± 12.67
LDL-C (mg/dL)	155.88 ± 23.41

Values are presented as mean ± SD, except for triglycerides which are expressed as median (P25, P75).

The baseline habitual dietary intake and physical activity levels of the subjects in the intervention and control groups are summarized in Table 3. No significant differences were observed between the groups regarding energy, macronutrient, or dietary fiber intake, nor in physical activity levels.

**Table 3:** Pre-intervention dietary intake and physical activity levels of the study subjects.

Parameters	Intervention group	Control group	*p-*value*
**Dietary intake**			
Energy (kcal/day)	1335 (880, 1583)	1518 (1027, 1744)	0.209
Carbohydrate (g/day)	151.26 (124.03, 189.18)	176.79 (120.11, 266.84)	0.344
Protein (g/day)	51.98 ± 18.55	57.66 ± 22.52	0.436
Fat (g/day)	48.37 (35.07, 58.71)	49.38 (33.36, 60.73)	0.642
Saturated fat (g/day)	11.71 (9.27, 15.49)	12.06 (9.64, 15.11)	0.850
Dietary fiber (g/day)	7.43 ± 3.60	6.72 ± 3.25	0.549
**Physical activity** (MET-min/week)	860 (340, 1560)	1040 (370, 2520)	0.448

Values are presented as mean ± SD or median (P25, P75).

*p-values were determined using the independent *t*-test for normal distributed data or the Mann Whitney *U* test for non-normally distributed data.

### 3.2 Gut microbiome responses to cricket powder supplementation

**Fig. 2:**
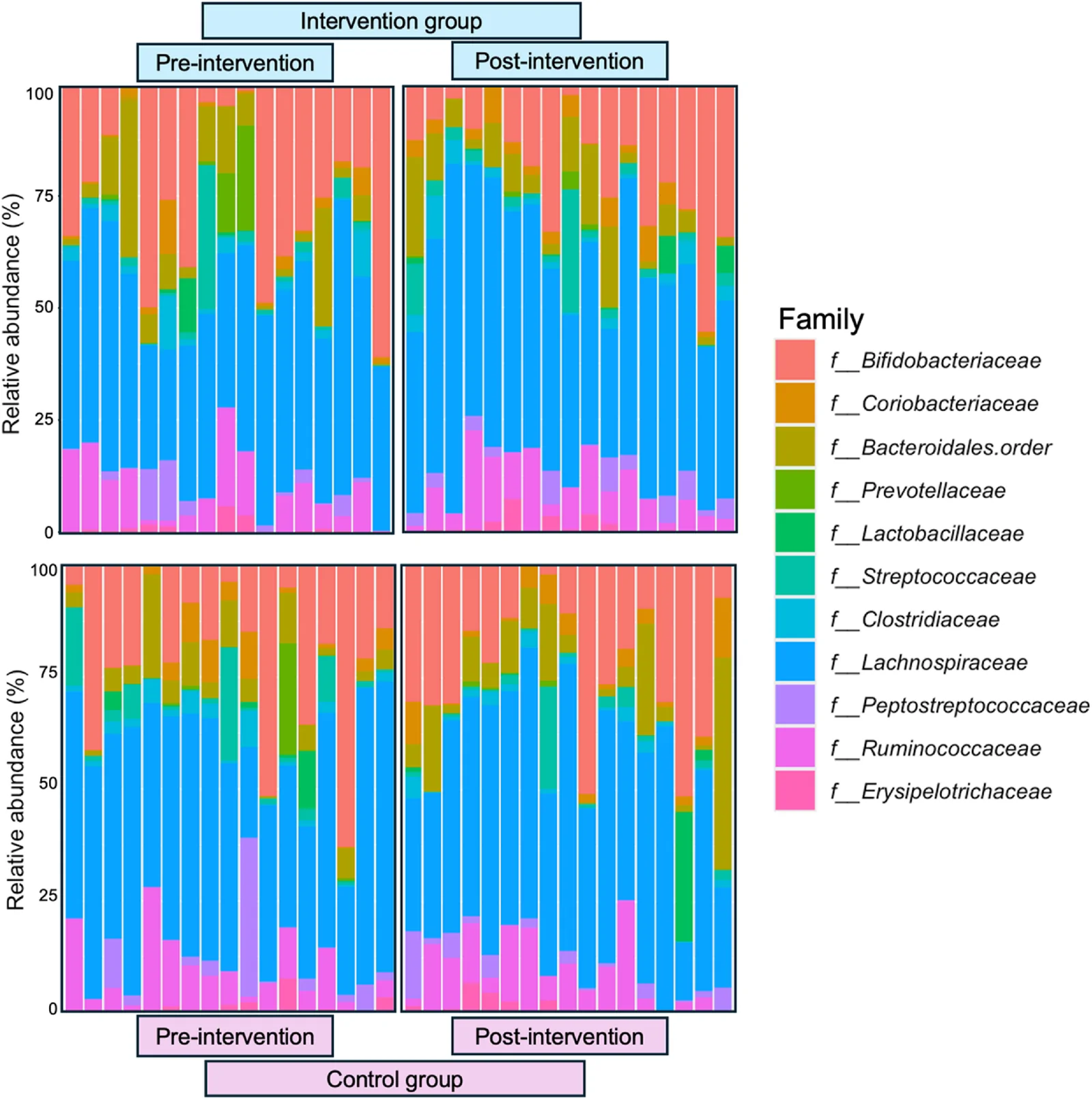
Gut microbiome composition at the family level across the intervention and control groups.

**Fig. 3:**
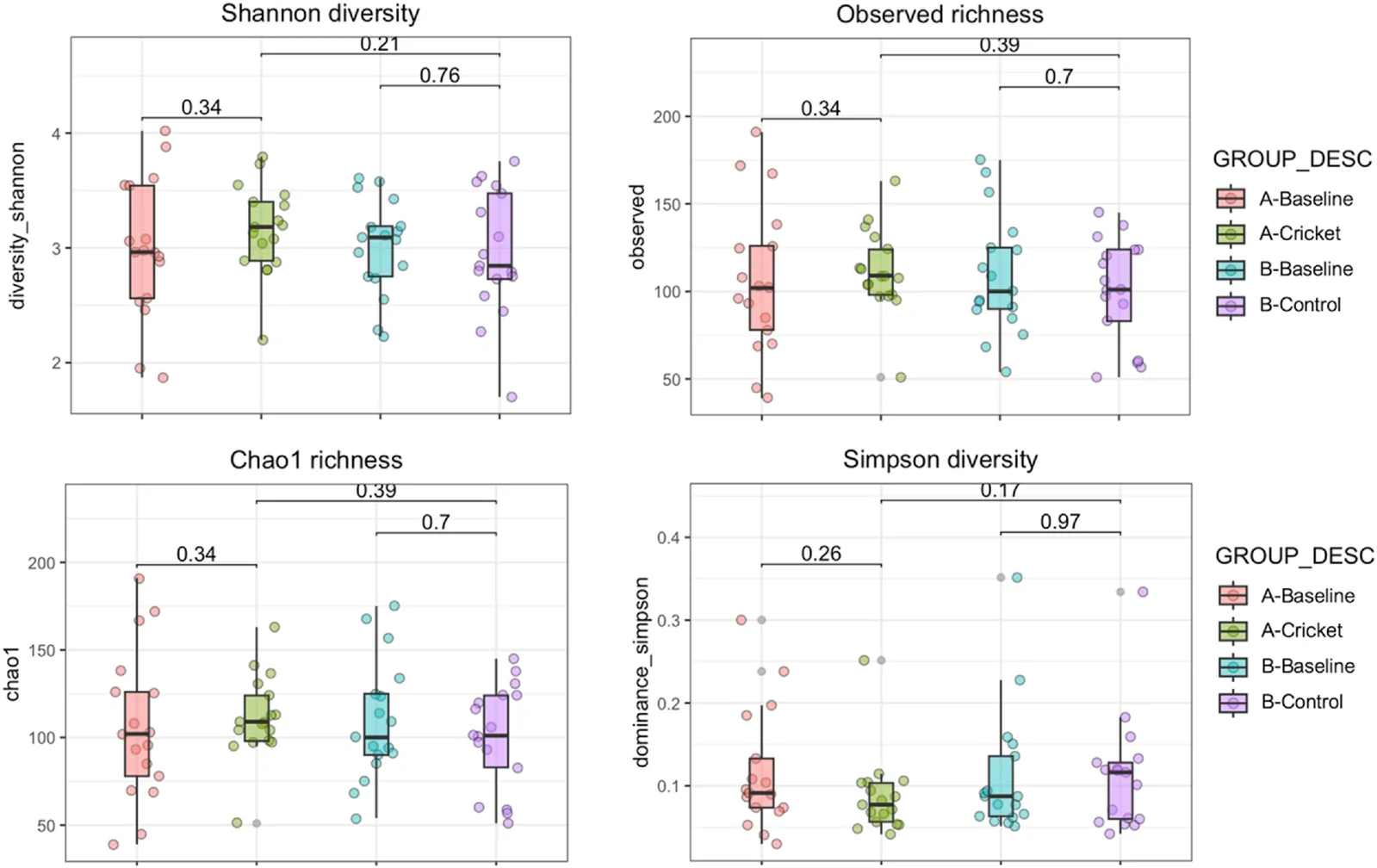
Alpha diversity of gut microbiome across the intervention and control groups. Groups are defined as follows: A-Baseline (pre-intervention state of the intervention group), A-Cricket (post-intervention state of the intervention group), B-Baseline (pre-intervention state of the control group), and B-Control (post-intervention state of the control group). *p*-values were obtained from the Wilcoxon signed-rank test for within-group comparisons and the Mann-Whitney *U* test for between-group comparisons.

**Fig. 4:**
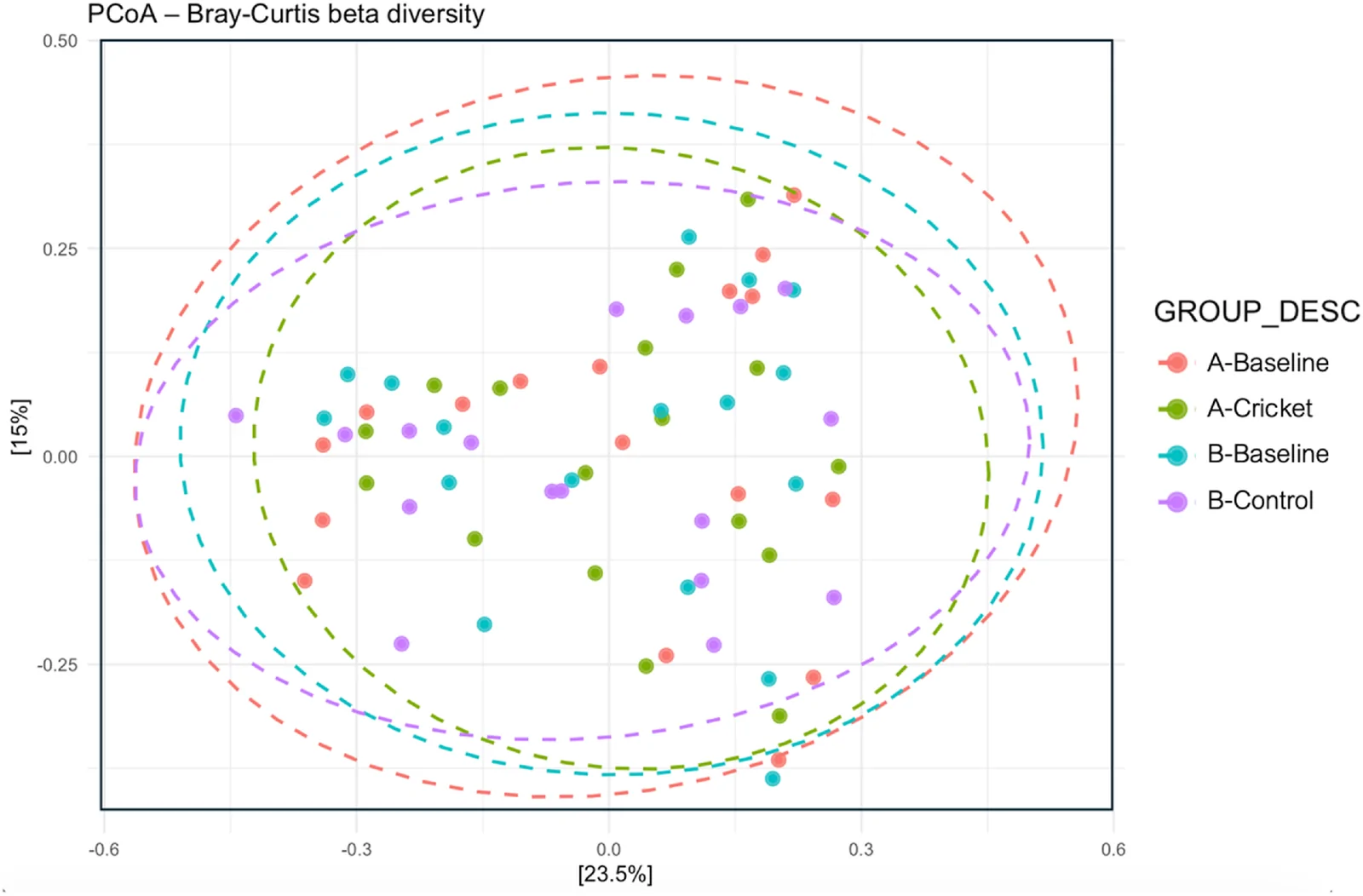
Beta diversity of gut microbiome based on Bray-Curtis dissimilarity. Groups are defined as follows: A-Baseline (pre-intervention state of the intervention group), A-Cricket (post-intervention state of the intervention group), B-Baseline (pre-intervention state of the control group), and B-Control (post-intervention state of the control group).

The overall gut microbiome composition remained stable between pre- and post-intervention and between intervention and control groups. At the family level, the microbial community was dominated by *Lachnospiraceae*, *Ruminococcaceae* and *Bifidobacteriaceae* (Fig. 2). Alpha diversity indices, including Shannon diversity, observed richness, Chao1 richness, and Simpson diversity, did not differ significantly across groups (Fig. 3). Similarly, Bray-Curtis beta diversity showed no clear clustering by intervention (Fig. 4), and PERMANOVA confirmed no significant difference in community structure among groups (R^2^ = 0.011, F = 0.24, *p* = 1.00).

**Fig. 5:**
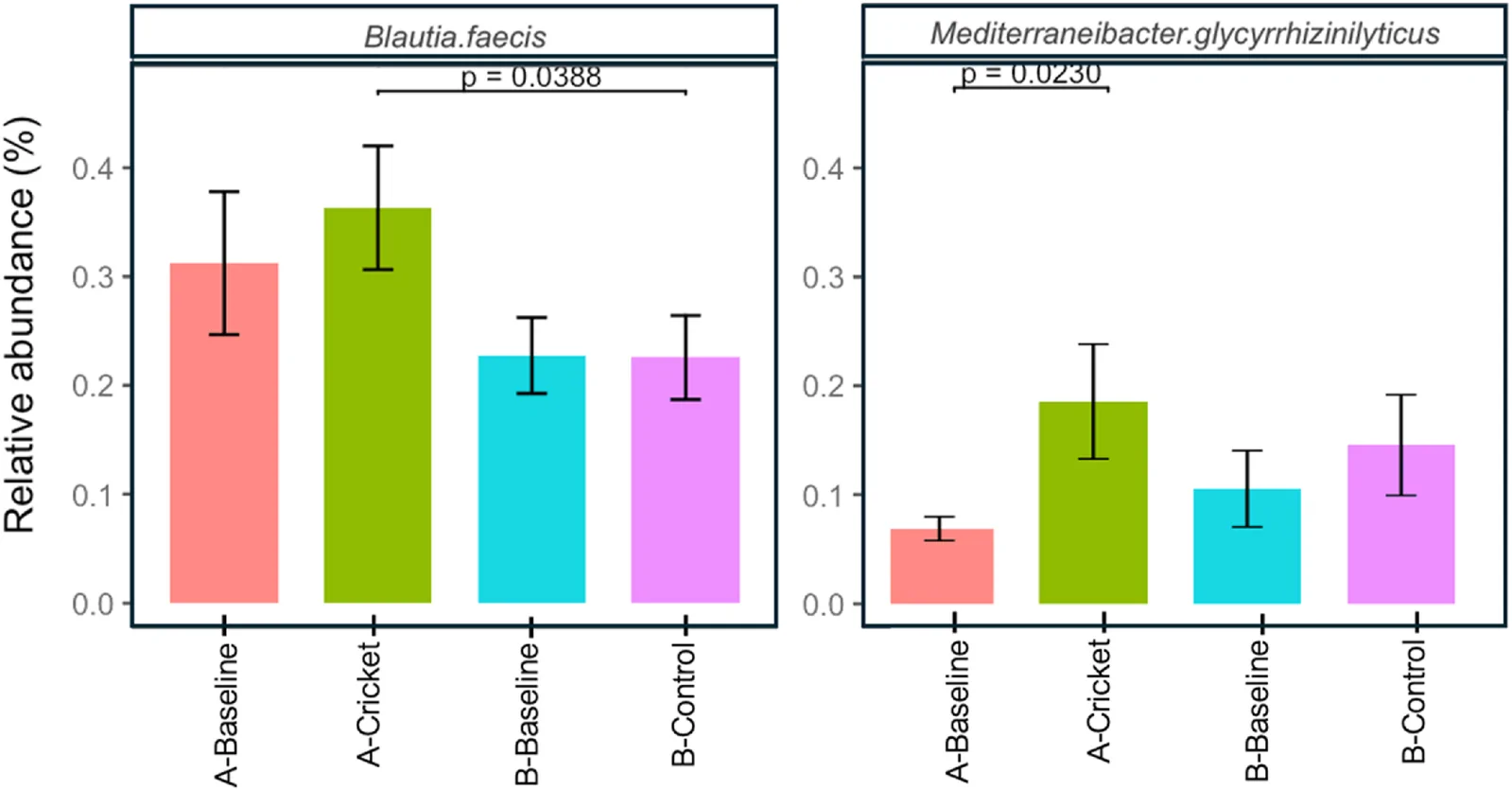
Relative abundance of significantly altered bacterial species across the intervention and control groups. Groups are defined as follows: A-Baseline (pre-intervention state of the intervention group), A-Cricket (post-intervention state of the intervention group), B-Baseline (pre-intervention state of the control group), and B-Control (post-intervention state of the control group). *p*-values were obtained from the Wilcoxon signed-rank test for within-group comparisons and the Mann-Whitney *U* test for between-group comparisons.

The relative abundance of significantly altered bacterial species across the intervention groups was analyzed, and the results are presented in detail in Table S2. Interestingly, at the species level, cricket powder supplementation significantly increased two species of *Lachnospiraceae*, namely *Blautia faecis* (*p* = 0.0388) and *Mediterraneibacter glycyrrhizinilyticus* (p = 0.0230) (Fig. 5). Changes in the relative abundance of bacterial species from pre-to post-intervention were calculated for each intervention period and compared between the intervention and control groups (Table 4). The change in *B. faecis* did not differ significantly between the two interventions (*p* = 0.306). In contrast, the change in *M. glycyrrhizinilyticus* differed significantly between the intervention and control groups (*p* = 0.038). These findings indicate that, after accounting for baseline differences, the observed change in *M. glycyrrhizinilyticus* differed between the two intervention periods, whereas no intervention-specific difference was observed for *B. faecis*.

**Table 4:** Post-intervention changes in bacterial relative abundance between study groups.

Bacterial species	Intervention group	Control group	P**
*Blautia faecis*	18.00 (−61.54, 102.92)	−15.95 (−84.00, 52.00)	0.306
*Mediterraneibacter glycyrrhizinilyticus*	24.32 (−0.45, 72.34)	1.30 (−23.80, 15.04)	0.038

Values are presented as median (P25, P75).

**P-values indicates between-group comparisons of the changes from pre-to post-intervention, determined using the Mann-Whitney *U* test.

### 3.3 Impact of cricket powder supplementation on fecal SCFA profiles

The SCFA profiles were compared between the intervention and control groups, as well as between pre- and post-intervention. No significant differences were observed in any SCFA levels within or between groups, as shown in Table 5. Acetic, butyric, and propionic acid levels showed only minor changes. Similarly, isovaleric and isobutyric acids also did not significantly increase in the intervention group.

**Table 5:** Effects of cricket powder supplementation on fecal SCFA profiles between the study groups.

SCFA (μmol/g fecal dry matter)	Intervention group		p*	Control group		p*	P**
	Pre	Post		Pre	Post		
Acetic acid	1.294 ± 0.508	1.232 ± 0.301	0.662	1.101 ± 0.363	1.144 ± 0.355	0.701	0.557
Butyric acid	0.732 ± 0.416	0.648 ± 0.410	0.492	0.598 ± 0.306	0.722 ± 0.505	0.363	0.253
Propionic acid	0.534 ± 0.262	0.552 ± 0.220	0.801	0.473 ± 0.206	0.549 ± 0.149	0.193	0.532
Isovaleric acid	0.060 (0.031, 0.146)	0.060 (0.035, 0.100)	0.687	0.048 (0.025, 0.126)	0.060 (0.045, 0.105)	0.124	0.284
Isobutyric acid	0.060 (0.035, 0.123)	0.070 (0.045, 0.095)	0.981	0.063 (0.030, 0.107)	0.070 (0.045, 0.125)	0.149	0.397

Values are presented as mean ± SD or median (P25, P75).

*p-values indicate within-group comparisons determined using the paired *t*-test for normally distributed data or the Wilcoxon signed-rank test for non-normally distributed data.

**P-values indicates between-group comparisons of the changes from pre-to post-intervention, determined using the independent *t*-test or Mann-Whitney *U* test.

### 3.4 Impact of cricket powder supplementation on blood lipid profiles

Blood lipid profiles showed no significant changes within or between the intervention and control groups. At pre-intervention, mean total cholesterol levels were 228.41 ± 26.09 mg/dL in the intervention group and 223.12 ± 24.43 mg/dL in the control group. Following the post-intervention, total cholesterol showed only minor, non-significant changes in both groups (intervention group: *p* = 0.160; control group: *p* = 0.165), with no significant difference between the groups (p = 0.645) (Table 6). Similarly, LDL-C, HDL-C, the LDL-C/HDL-C ratio, and triglyceride levels remained stable without significant changes. Post-intervention, LDL-C showed slight, non-significant increases in both groups (intervention group: *p* = 0.395; control group: p = 0.306). Meanwhile, HDL-C showed a small increase in the intervention group, but this change did not reach statistical significance (*p* = 0.066). The LDL-C/HDL-C ratio and median triglyceride levels also remained unchanged throughout the study period. Overall, cricket powder supplementation for 21 days did not significantly alter blood lipid profiles.

**Table 6:** Effects of cricket powder supplementation on blood lipid levels across the intervention and control groups.

Parameters	Intervention group		p*	Control group		p*	P**
	Pre	Post		Pre	Post		
Total cholesterol (mg/dL)	228.41 ± 26.09	238 ± 25.10	0.160	223.12 ± 24.43	229.12 ± 21.83	0.165	0.645
HDL-C (mg/dL)	56.00 ± 11.60	58.82 ± 11.10	0.066	57.18 ± 12.20	57.35 ± 10.40	0.914	0.229
LDL-C (mg/dL)	158.94 ± 20.56	164.29 ± 27.05	0.395	154.29 ± 22.92	158.41 ± 20.94	0.306	0.866
LDL-C/HDL-C ratio	2.96 ± 0.75	2.90 ± 0.77	0.625	2.82 ± 0.75	2.86 ± 0.67	0.749	0.558
Triglyceride (mg/dL)	94.00 (68.00, 157.00)	95.00 (79.00, 128.00)	0.448	85.00 (65.00, 119.50)	83.00 (71.00, 113.00)	0.795	0.836

Values are presented as mean ± SD or median (P25, P75).

*p-values indicate within-group comparisons determined using the paired *t*-test for normally distributed data or the Wilcoxon signed-rank test for non-normally distributed data.

**P-values indicates between-group comparisons of the changes from pre-to post-intervention, determined using the independent *t*-test or Mann-Whitney *U* test.

### 3.5 Defecation frequencies and gastrointestinal tolerance during cricket powder supplementation

**Fig. 6:**
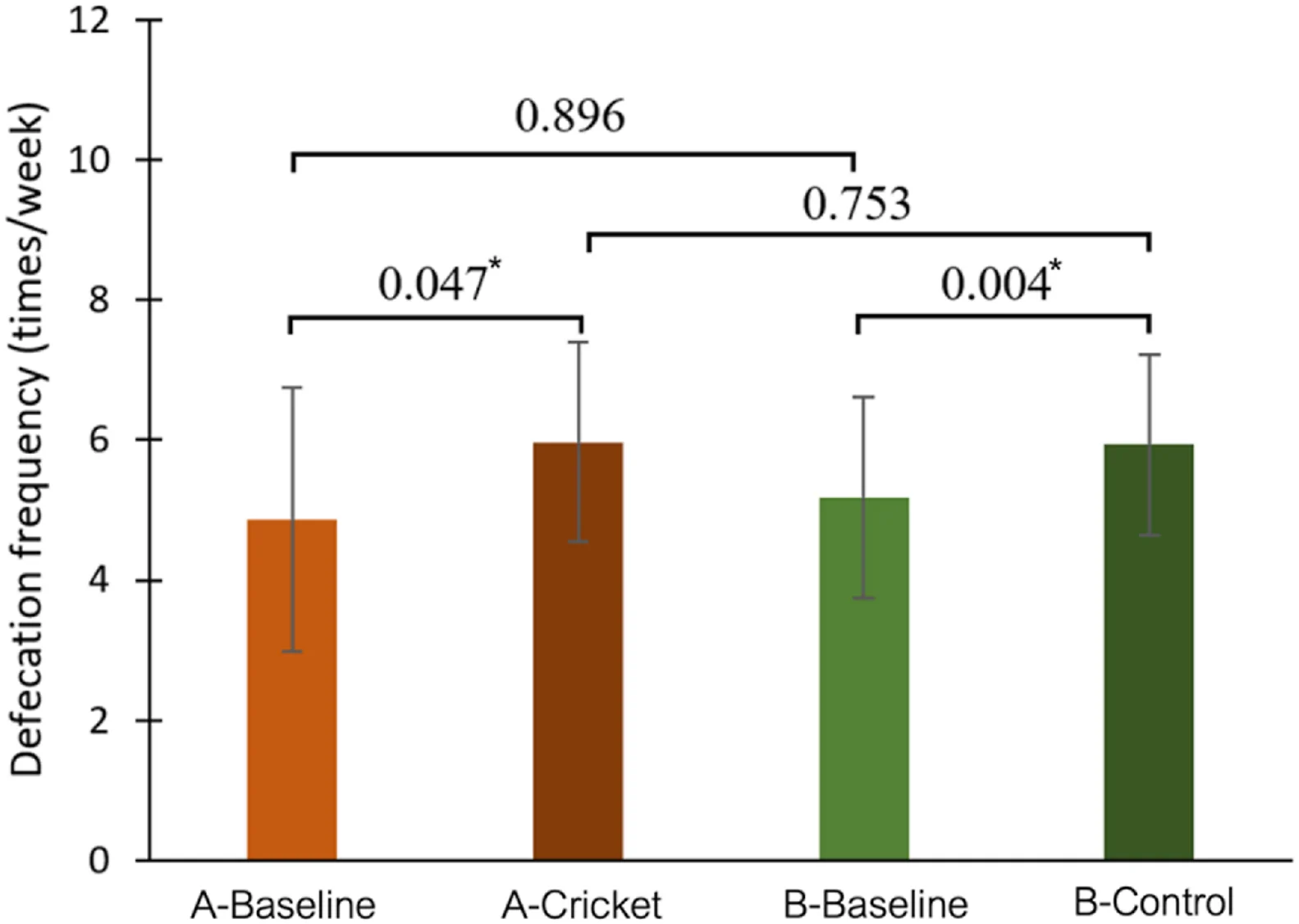
Defecation frequency of the study subjects at pre- and post-intervention. Groups are defined as follows: A-Baseline (pre-intervention state of the intervention group), A-Cricket (post-intervention state of the intervention group), B-Baseline (pre-intervention state of the control group), and B-Control (post-intervention state of the control group). *p*-values were obtained from the Wilcoxon signed-rank test for within-group comparisons and the Mann-Whitney *U* test for between-group comparisons.

Defecation frequencies, as recorded in subjects’ diaries throughout the intervention period, were similar at pre-intervention state. As shown in Fig. 6, a significant increase in defecation frequency was observed in both intervention and control groups. Stool characteristics showed only minimal changes in both intervention and control groups. Overall, most subjects maintained normal stool consistency after both interventions. Stool quantity, color, and odor remained within normal or acceptable ranges, with only minor individual variations observed. These findings suggest that cricket powder supplementation did not cause clinically meaningful changes in bowel characteristics.

## 4 Discussion

Edible crickets have gained attention as a sustainable, nutrient-dense protein source with potential prebiotic properties. While previous studies suggest that cricket powder may promote SCFA-producing bacteria, evidence remains limited, particularly regarding its effects on gut microbiome composition and lipid metabolism in Asian populations. In this randomized crossover controlled trial in Thai subjects with high or borderline-high LDL-C, we comprehensively evaluated gut microbiome responses, fecal SCFA profiles, and blood lipid profiles following 21 days of cricket powder supplementation.

The results of this study showed that cricket powder supplementation did not alter the overall diversity or global structure of the gut microbiome. However, it modulated specific bacterial species. Notably, an increase was observed in beneficial commensal bacteria such as *B*. *faecis* (family Lachnospiraceae), a species known for acetate-producing capacity and contribution to host energy metabolism and immune regulation (Verstraeten et al., 2022). In addition, enrichment was detected in glycan-utilizing bacterium, *M. glycyrrhizinilyticus* (Lachnospiraceae). This taxon is recognized for their ability to degrade complex carbohydrates and plant- or host-derived glycans, thereby contributing to carbohydrate fermentation in the colon (Sakuma et al., 2006). This pattern of selective modulation by cricket powder is consistent with previous findings from prebiotic interventions, such as copra meal hydrolysate (CMH) (Sathitkowitchai et al., 2021) and chitin-glucan (Ranaivo et al., 2022). These observed effects may be attributable to the presence of chitin and chitin-derived oligosaccharides in cricket powder. Previous study has reported that cricket-derived chitosan exhibits prebiotic-like properties by promoting the growth of probiotic bacteria, including *Lactobacillus fermentum*, *Lactobacillus acidophilus*, and *Bifidobacterium adolescentis* (Kipkoech et al., 2021).

However, unlike the findings reported by Stull et al., 2018) (Stull et al., 2018), this present study did not demonstrate a bifidogenic effect induced by cricket powder, despite the presence of *Bifidobacteriaceae* in the study population. This discrepancy may be explained by population-specific microbiome characteristics. In Thai adults, the gut microbiome is enriched in members of *Lachnospiraceae* and *Ruminococcaceae*, which possess extensive repertoires of carbohydrate-active enzymes and transport systems for complex carbohydrate metabolism (Raethong et al., 2021). This baseline microbial profile may therefore favor the early expansion of glycan-degrading taxa, including *Lachnospiraceae* and *Ruminococcaceae*, before a clear bifidogenic response becomes evident. Nevertheless, the selective enrichment of potentially beneficial bacteria, including *B*. *faecis* and *M. glycyrrhizinilyticus* seen in this study, together with the absence of pathogenic bacterial overgrowth, may supports the potential of cricket powder as a prebiotic.

Moreover, the gut microbiome functions as an important metabolic interface between diet and host physiology, supporting nutrient utilization and energy metabolism through continuous metabolite exchange with host (Sung et al., 2017; LeBlanc et al., 2017). Among microbial metabolites, SCFAs are among the most studied because they contribute to immune regulation, metabolic homeostasis, and intestinal barrier function (den Besten et al., 2013; Furusawa et al., 2013). In the present study, no significant differences in SCFA concentrations were observed following 21 days of cricket powder supplementation, either within or between groups. Levels of major SCFAs, including acetic acid, propionic acid and butyric acid exhibited only minor changes. Similarly, branched-chain SCFAs such as isovaleric and isobutyric acids, typically derived from protein fermentation of branched-chain amino acids (Smith and Macfarlane, 1998), did not show significant increases. The absence of elevated branched-chain SCFAs suggests that cricket powder supplementation did not promote excessive protein fermentation in the colon, which is often associated with the production of potentially harmful metabolites and intestinal irritation. Consistent with this, no significant differences in SCFA profiles were observed between the intervention and control groups, indicating that cricket powder supplementation did not disrupt gut microbial metabolic balance or induce adverse gastrointestinal effects. Importantly, the maintenance of butyrate levels further suggests that intestinal barrier function and anti-inflammatory capacity were not negatively affected. These findings are also consistent with gastrointestinal tolerance outcomes, as subjects generally reported well-formed stools and stable bowel habits, without symptoms of constipation or diarrhea. Stool characteristics, including volume, color, and odor, remained largely unchanged. Although a slightly stronger odor was reported in some subjects following cricket powder supplementation, this may be attributed to increased protein fermentation, which produces sulfur-containing gases (Hansen and Venkatachalam, 2023). Notably, no increase in sulfate-reducing bacteria or branched-chain SCFAs was detected, suggesting that such effects were minimal and not indicative of dysbiosis.

Regarding metabolic outcomes, no significant changes were observed in total cholesterol or LDL-C levels following cricket powder supplementation. However, a trend toward increased HDL-C was noted, while the LDL-C/HDL-C ratio remained unchanged. These findings suggest that cricket powder consumption did not adversely affect cardiovascular risk profiles (Li et al., 2024).

From a food safety perspective, an important finding of the present study is that daily supplementation with cricket powder was generally well tolerated by the subjects. Although defecation frequency increased following both the cricket and control groups, stool characteristics, including consistency, quantity, color, and odor, showed only minimal changes and remained within generally acceptable ranges. These findings suggest that supplementation of 21.5 g/day of cricket powder for 21 days did not result in clinically meaningful alterations in bowel characteristics. From a practical perspective, gastrointestinal tolerability is an important consideration when incorporating edible insects into habitual diets. Overall, the acceptable tolerability of cricket powder supplementation, together with the integrated assessment of clinical, microbial, and metabolic outcomes, provides preliminary support for further investigation of edible insects as sustainable functional food ingredients.

While the crossover design minimized inter-individual variability and reduced potential confounding effects and a comprehensive set of biomarkers, including gut microbiome, SCFAs, blood parameters and gastrointestinal assessments, provided evidence supporting the functions of cricket powder. Nevertheless, some limitations should be considered. Firstly, subjects were not exposed to the taste of cricket powder prior to the intervention, leading to some dropouts due to acceptability issues. Secondly, we acknowledge that the control product was not isocaloric with the cricket-powder porridge. However, our study was designed as a food-based intervention, in which cricket powder was added to a commonly consumed rice porridge and compared with the same porridge without cricket powder. We therefore interpreted the dietary intake, microbiome, and metabolic outcomes with appropriate caution. Future studies should include a nutrient-matched comparator to better isolate the specific effects of cricket powder. Finally, the short intervention period and small sample size may limit the generalizability of the findings. Future studies with larger cohorts and longer durations are warranted to confirm these results and evaluate long-term health effects.

## 5 Conclusion

Cricket powder supplementation in Thai subjects with high or borderline-high LDL-C resulted in selective modulation of gut microbiome, characterized by enrichment of glycan-degrading and potentially beneficial bacterial species, without altering overall microbial diversity or SCFA production. The absence of increased branched-chain SCFAs and pathogenic bacterial overgrowth indicates that cricket powder does not promote excessive proteolytic fermentation or microbial imbalance. Moreover, stable gastrointestinal tolerance and unchanged lipid profiles further support its short-term safety and metabolic homeostasis.

## Data statement

Data will be made available on request.

## Author contributions

Aree Prachansuwan: Writing – original draft, Methodology, Investigation, Validation, Data curation, Writing – review & editing. Pitchayaporn Sukkha: Methodology, Investigation, Formal analysis. Parunya Thiyajai: Methodology, Investigation, Validation, Data curation. Pitakthai Chamtim: Methodology, Investigation. Sarunya Kitdumrongthum: Methodology. Pimnapanut Sridonpai: Methodology. Angkansiri Dee-uam: Methodology. Karaked Tongdonpo: Methodology. Dunyaporn Trachootham: Supervision. Warangkana Srichamnong: Supervision. Iyarit Thaipisuttikul: Supervision. Nachon Raethonga: Writing – original draft, Supervision, Methodology, Investigation, Validation, Conceptualization, Data curation, Visualization, Project administration, Writing – review & editing, Funding acquisition.

## Ethics declaration

Written informed consent to take part in the study and to publish the article has been obtained from all participants or their legal representatives. The privacy rights of participants have been observed.

This study was performed in compliance with relevant laws, regulatory frameworks and guidelines where the research took place. This study was approved by the Mahidol University Central Institutional Review Board. (Approval No. COA No. MU-MOU 2023/334.2710)

This clinical trial was registered with number NCT07009756 (ClinicalTrials.gov).

## Funding

This research project has been supported by 10.13039/501100004156Mahidol University (Fundamental Fund: fiscal year 2025 by National Science Research and Innovation Fund (NSRF)).

## Declaration of competing interests

The authors have no declaration of interest.
